# Development of Sensitive Methods for the Detection of Minimum Concentrations of DNA on Martian Soil Simulants

**DOI:** 10.3390/life13101999

**Published:** 2023-09-30

**Authors:** Yongda Li, Keith D. Rochfort, David Collins, Konstantinos Grintzalis

**Affiliations:** 1 School of Biotechnology, Dublin City University, D09 Y5NO Dublin, Ireland; yongda.li29@mail.dcu.ie (Y.L.); david.collins@dcu.ie (D.C.); 2 School of Nursing, Psychotherapy, and Community Health, Dublin City University, D09 Y5NO Dublin, Ireland; keith.rochfort@dcu.ie

**Keywords:** DNA, quantification, assays, intercalators, Hoechst, SYBRGreen, NovelJuice, Martian simulants, biosignatures

## Abstract

Several methods used for the quantification of DNA are based on UV absorbance or the fluorescence of complexes with intercalator dyes. Most of these intercalators are used in gels to visualize DNA and its structural integrity. Due to many extraterrestrial samples, such as meteorites or comets, which are likely to contain very small amounts of biological material, and because the ability to detect this material is crucial for understanding the origin and evolution of life in the universe, the development of assays that can detect DNA at low limits and withstand the rigors of space exploration is a pressing need in the field of astrobiology. In this study, we present a comparison of optimized protocols used for the fast and accurate quantification of DNA using common intercalator dyes. The sensitivity of assays exceeded that generated by any commercial kit and allowed for the accurate quantification of minimum concentrations of DNA. The methods were successful when applied to the detection and measurement of DNA spiked on soil samples. Furthermore, the impact of UV radiation as a harsh condition on the surface of Mars was assessed by DNA degradation and this was also confirmed by gel electrophoresis. Overall, the methods described provide economical, simple-step, and efficient approaches for the detection of DNA and can be used in future planetary exploration missions as tests used for the extraction of nucleic acid biosignatures.

## 1. Introduction

The ExoMars 2022 Mission aims to analyse the traces of organic material from samples acquired at a depth of 2 m under the surface of Mars. The overarching objective of this mission is to determine whether the organic material contains evidence of past life or conditions that could support life on the planet [1,2]. Thus, it is essential to develop direct and sensitive methods for detecting life signals, which can be performed by instruments to allow the in situ biosignature detection on Mars. While it is unknown whether extra-terrestrial life will resemble life on Earth, it is important to start searching for biosignatures using Earth as a reference for extra-terrestrial life [3].

As the essential component of life that carries the genetic information of organisms, DNA could be considered a valuable category of biomolecules for investigation in future space missions [4]. However, due to the harsh conditions on the surface of Mars, which include UV radiation and thin atmosphere, biological macromolecules, such as DNA, are subject to varying degrees of damage and degradation [5]. Hence, in order to detect potential DNA under these harsh conditions, it is necessary to develop robust assays that are capable of detecting DNA at lower limits.

Studies focusing on life on Mars and the meteorite transfer between Mars and Earth, showed that nucleic acids, as biological informational polymers, could be a worthwhile research subject to prove the existence of life on Mars [6]. It has also been proved that the possibility of an in situ nucleic acid extraction and detection via nanopore sequencing could be feasible [6]. Contrary to a common perception, advancements in in situ technologies for the detection of charged genetic polymers, including DNA, on Mars are currently underway. One such notable initiative is the development of an Agnostic Life Finder (ALF) instrument, designed for the large-scale screening of Martian life during in situ refueling, as highlighted in the study by Špaček and Benner (2022) [7] and the ALFA Mars initiative. Nonetheless, traditional analytical techniques also have their merits. Gas chromatography–mass spectrometry (GC-MS) is a powerful analytical technique which is widely used for the detection and identification of organic compounds in various samples with high sensitivity and selectivity [8]. For example, the Curiosity rover uses pyrolysis–gas chromatography–mass spectrometry (py-GC-MS) to detect organic compounds from samples collected on Mars, which could be also used for detecting DNA [9]. Raman spectroscopy is another analytical technique utilized in Mars rovers that identifies chemical compositions through the analysis of molecular vibrations, offering additional avenues for the detection of biomolecules, including DNA [10].

DNA analysis typically involves gel electrophoresis in laboratory settings, while the accurate quantification of the concentration of DNA is usually based on ultraviolet (UV) absorbance, fluorescence staining, and methods that require a chemical reaction (such as a diphenylamine reaction) [11]. However, UV measurements are not sensitive and additional sensitivity can be achieved with fluorescent intercalator dyes, such as Hoechst, acridine orange [12,13], methylene blue [14,15], and SYBRGreen [16]. These fluorescent dyes create complexes with DNA fragments and fluoresce differently depending on the size of the DNA fragments, with fragmented DNA fluorescing being up to 70% less than intact DNA samples [17,18]. In one particular study which utilized PicoGreen for the quantitative assessment of fragmented and nicked dsDNA, it was demonstrated that the sensitivity of such methods can detect as little as 5 pg of DNA [19]. Thus, it can be employed with exceptional precision, effectively detecting low concentrations of DNA with a high degree of accuracy. An additional benefit of this approach is its relative simplicity, which obviates the need for extensive sample preparation or processing [20]. Especially in challenging conditions and with limited resources of a Mars mission, this technique is highly amenable to deployment. Moreover, the utilization of UV measurements with fluorescent dyes allows for the remote detection of DNA, with the potential for deployment via a portable or handheld UV spectrophotometer [21]. This innovative approach could facilitate on-site testing for DNA by astronauts or rovers, thereby circumventing the need to transport samples back to Earth for further examination.

In a number of previous studies, we applied this principle to identify the fragmentation status of DNA samples and developed sensitive protocols for their application [19] in in vitro systems [22]. In this study, we optimized protocols for the quantification of DNA using common fluorescent dyes and compared them for their efficiency. Thereafter, we optimised the spiking and recovery of DNA from a Martian soil simulant and successfully evaluated the extraction of DNA from this complex sample using agarose gel electrophoresis and the impact of harsh environmental conditions inherent to the surface of Mars, such as UV radiation, on DNA structure and concentration. With the increasing interest in Mars exploration and the potential search for biosignatures, there is a growing need for sophisticated yet adaptable methodologies that can be integrated into Martian rovers. Such methods should not only be sensitive and accurate but also compatible with the operational constraints of these rovers. Ultimately, this study could contribute to the development of sensitive and reliable methods for detecting biosignatures on Mars, paving the way for future exploration and the potential discovery of extra-terrestrial life.

## 2. Materials and Methods

### 2.1. Materials

JSC-Mars 1: In this study, JSC-Mars 1Mars Soil Simulant was utilized as a soil sample to simulate the soil of Mars. Martian soil is predominantly composed of reddish-brown, windblown dust with a particular particle size distribution. For the purposes of this study, altered volcanic ash from Hawaii was selected as a suitable analogue to Martian soil, due to its similarity in both colour and particle size. Specifically, the JSC-Mars 1Mars Soil Simulant was utilized as a proxy for Martian soil to facilitate the testing of the proposed methodology for in situ DNA detection on Mars.

### 2.2. DNA Preparation and Fluorescence Measurements

Double-stranded DNA from salmon testes (Sigma Aldrich, St. Louis, MO, USA, D1626) was dissolved in sterile Tris-EDTA (TE) solution (pH 7.4) (from Fisher Scientific, Ireland) overnight at 4 °C at a concentration of 2 mg DNA per mL. Additionally, λ phage DNA (Fisher Scientific) was also prepared in sterile TE solution as a different size of dsDNA. DNA standards were diluted in sterile phosphate buffer saline (PBS, from Fisher Scientific) and 0.2 mL was mixed with 0.05 mL of different dyes (as described in the assay development section). For Hoechst dye, fluorescence was measured at an excitation/emission wavelength of 360/460 nm; for Novel Juice, it was measured at 485/535 nm; and for PicoGreen, it was measured at 485/535 nm using a microplate reader (Tecan Trading AG). Appropriate reagent blanks were prepared and the linear standard curves were plotted to confirm the detection limit and concentrations of unknown analysed samples. The optimised fluorescence measurement conditions for all dyes were selected based on the background and maximum sensitivity achieved (as described in the Results section).

### 2.3. DNA Spiking on Soil Samples and Recovery

**Figure 1:**
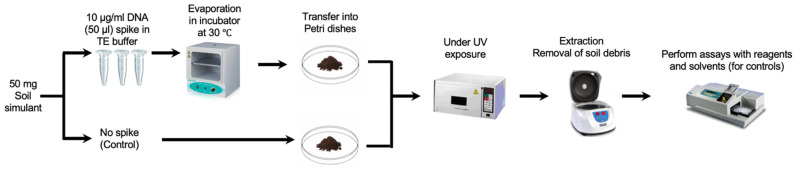
Spiking and recovery of DNA samples (the execution of DNA detection assays using specific reagents). Parallel controls are run using only the solvent without the presence of any reagents for comparative analysis.

The DNA was prepared in sterile TE solution and spiked on the JSC Mars-1A Martian Regolith Simulant, which was kindly provided by the NASA Johnson Space Centre. The DNA was then extracted in sterile PBS and the extract was split in two portions, one of which was completely fragmented by sonication and measured against the other to validate the DNA signal. To simulate the harsh conditions more accurately on the surface of Mars, our initial experiments focused on the impact of UV radiation on the stability of DNA in soil simulants. Based on measurements taken by the Curiosity rover, the total UV radiation dosage at Gale Crater was estimated to be no more than 20 W/m^2^, or 72 kJ/hm^2^ [23]. Accordingly, the spiked soil samples were exposed to the same UV dosage of 72 kJ/hm^2^ to provide a more representative Martian condition (Figure 1). The optimised conditions for spiking and recovery were performed using the Hoechst dye (as described in the Results section). However, it is important to note that Martian soil also contains a range of oxidizing substances, such as perchlorates and hydrogen peroxide [24]. Future experiments will need to incorporate these factors to better simulate the Martian environment.

### 2.4. DNA Fragmentation Assessment with Gel Electrophoresis

The fragmentation status of DNA (by UV or sonication) was validated with a 3% concentration of agarose gel electrophoresis. In total, 3 g of ultra-pure agarose (Sigma Aldrich) was dissolved in 100 mL of 1× Tris acetate-EDDTA (TAE) buffer. The mixture was gently brought to a boil until the solution had turned clear and the agarose had fully dissolved. Once cooled to 50–60 °C, 10 μL of SYBRSafe was added to the solution and mixed. The mixture was then poured into casting plate and left for 20 min until the gel fully polymerized. Once set, the gel was placed into the gel rig and submerged with the addition of 1× TAE. Prior to loading onto the gel, each DNA sample was mixed with 4× Loading buffer (40% w/v sucrose, 0.25% w/v bromophenol blue). The comb was removed and the samples were then added into the preset lanes. After sample loading, 6 μL of GeneRuler 100 bp DNA Ladder Plus was added for relative band size comparison. The gel was then resolved for ~90 min at 100 V before being visualized using a G-BOX gel documentation and analysis system.

## 3. Results

### 3.1. Optimisation of the Fluorescence Measurements of DNA Complexes with Intercalator Dyes

**Figure 2:**
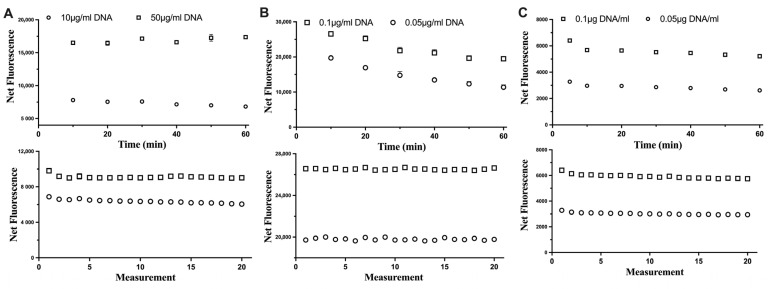
The impact of the incubation time (top row) and consecutive excitation (bottom row) of DNA with (**A**) Hoechst, (**B**) Novel Juice, and (**C**) PicoGreen dyes. Data represent average ± SD (N = 3).

The stability of each fluorescent dye was assessed in relation to the incubation time with DNA samples and consecutive excitations of DNA-dye complexes (Figure 2). For all three fluorescent dyes tested, the consecutive excitation of the same DNA sample did not impact its fluorescence. This would allow the assay to measure samples (if needed) repeatedly. However, the reaction of Hoechst and DNA could be stable up to 60 min, while for the Novel Juice, the fluorescence signal was stable for only 10 min and then started to decrease, which indicated that the optimum incubation time should not exceed 10 min for this dye. For PicoGreen, the fluorescence signal had a small decrease at the first 10 min and then was maintained stable. This indicated that the time window for incubating and measuring the fluorescence using PicoGreen can be even up to 60 min.

**Figure 3:**
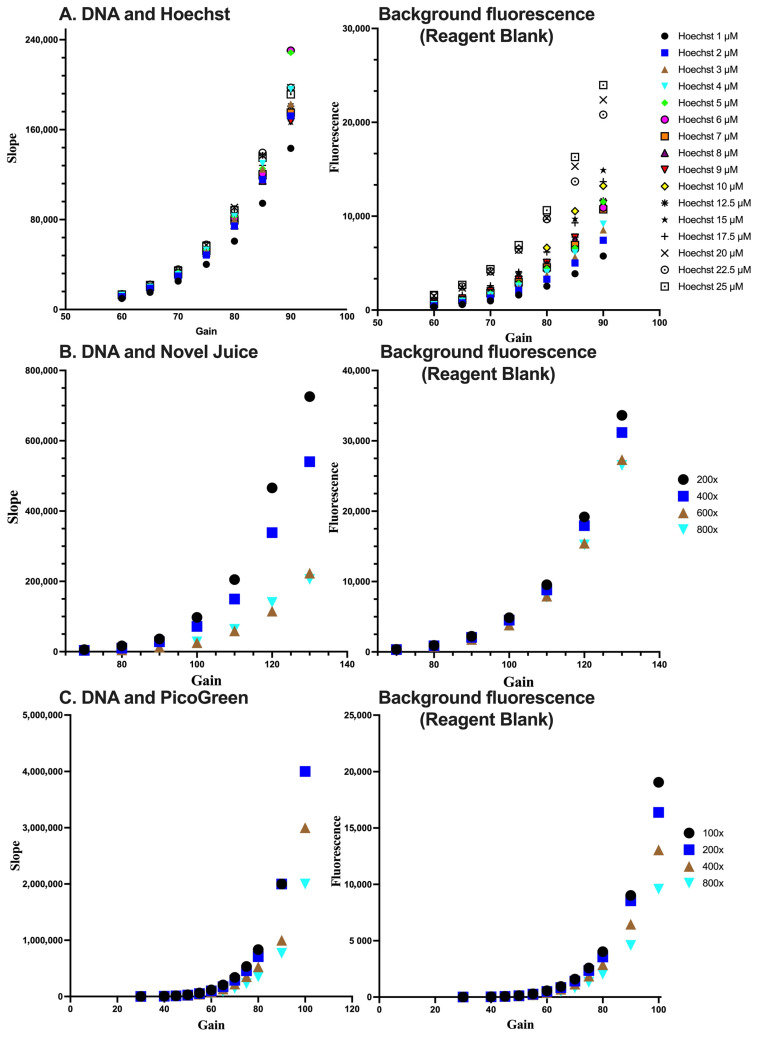
Optimisation of the reagent concentration and sensitivity of measurements for (**A**) Hoechst, (**B**) Novel Juice, and (**C**) PicoGreen. For (**B**,**C**), numbers indicate the dilution of the initial reagent. Data represent average ± SD (N = 3).

In order to optimize conditions for the detection of DNA, the DNA samples were subjected to measurements at different concentrations of each fluorescent dye. Subsequently, the combination of dye concentration and the gain of the fluorescent plate reader, which is indicative of the sensitivity of the fluorescence signal measurement, was determined. Through this process of identifying the ideal detection conditions for DNA, the accuracy and reliability of the experimental results can be enhanced. The sensitivity for each dye was assessed by the linear standard curve (Figure 3). In each case, the optimum condition for Hoechst was at a 5 µM concentration and a gain value set at 70%. For Novel Juice and PicoGreen, the optimum dilution of the commercial stock of reagents was 400-fold and the gain values were set at 100% and 80%, respectively. Increasing the sensitivity of the fluorescence measurement results in a desired increase in the method’ sensitivity, as expressed by the slope of the standard curve; however, the background of the reagent blank also increases. Therefore, deciding the optimum conditions for each of the dyes was a compromise between the background fluorescence and the maximum sensitivity, which is near the inflection point of the parabolic trends observed in Figure 2 for the different dyes.

**Figure 4:**
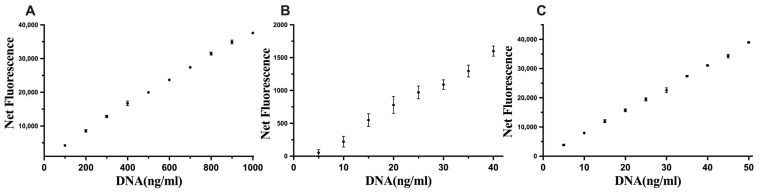
Linear standard curves for (**A**) Hoechst, (**B**) Novel Juice, and (**C**) PicoGreen. Data represent average ± SD (N = 4).

The optimised conditions were assessed with DNA standards, and the sensitivity limits were 100–1000 ng of DNA/mL, 5–40 ng of DNA/mL, and 5–50 ng of DNA/mL for Hoechst, Novel Juice, and PicoGreen, respectively (Figure 4), which improved upon the commercial kit results for the quantification of DNA.

### 3.2. Spiking and Extraction of DNA from Martian Soil Simulants and the Impact of UV Radiation

**Figure 5:**
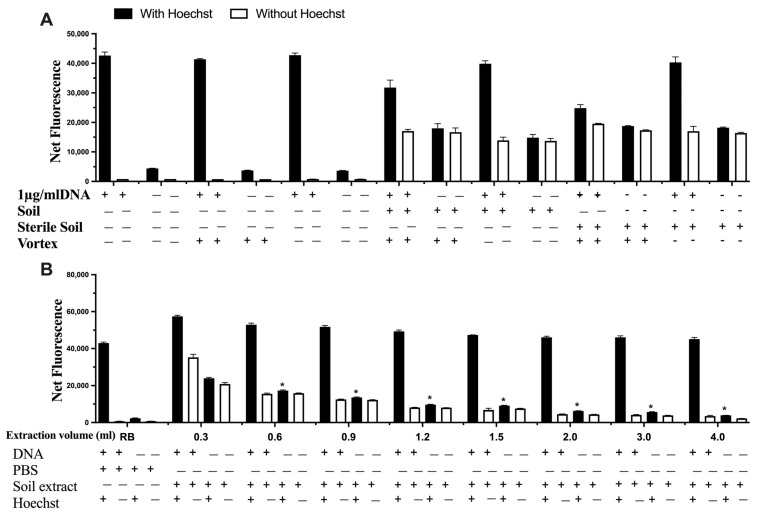
The impact of (**A**) sterile soil and (**B**) the extraction volume on signal interference on detecting DNA. Data represent average ± SD (N = 4). RB: Reagent Blank (for control). * Statistically significant based on two-way ANOVA corrected with the post hoc Tukey test.

The interference of the soil itself was explored by autoclaving the soil prior to mixing it with DNA (Figure 5A), which was subsequently spiked with DNA and extracted using different volumes of sterile phosphate buffer saline (PBS, Figure 5B). The results indicate that there was no difference using sterile soil or non-sterile soil, thus indicating no degradation or additional DNA from the soil. In relation to the extraction step, the increase in extraction volume significantly decreased possible interferences from the soil matrix (Figure 5B).

**Figure 6:**
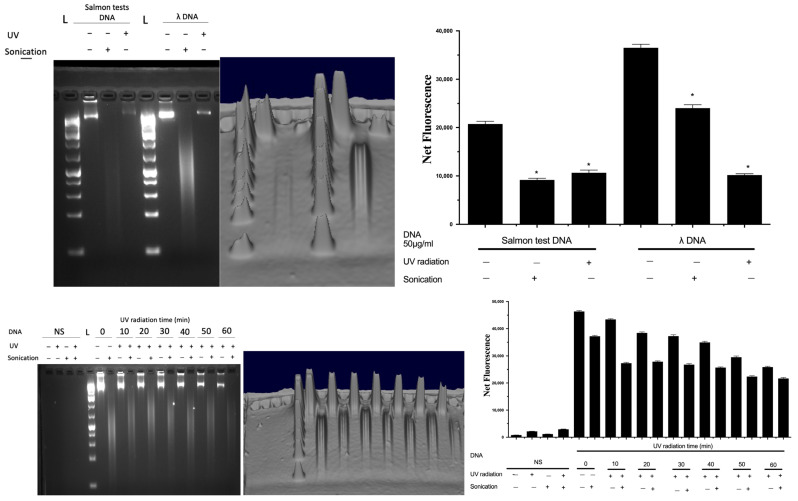
Quantification of DNA samples and evaluation of their fragmentation status. Data represent average ± SD (N = 4). NS: non-spiked soil, L: DNA ladder, Net FU: net fluorescence unit. * Statistically significant based on two-way ANOVA corrected with the post hoc Tukey test.

To validate the fluorescent signal that originated solely from complexing with DNA and not from any other material, sonication was used as a control to completely fragment the extracted DNA, since fragmentation would result in a decrease in the fluorescence signal from DNA [17,19]. Furthermore, the impact of UV on DNA spikes in soil was confirmed using agarose gel electrophoresis (Figure 6). DNA was spiked into soil, which was subsequently exposed to UV for an hour, and subsequently extracted in sterile PBS and subjected to sonication and analysed by gel electrophoresis (Figure 6). DNA from salmon testes or bacteriophage λ was used as a representative nucleic acid, and UV radiation resulted in the breakdown of intact DNA, which was also verified by both the decrease in fluorescence (by Hoechst) and gel electrophoresis. A time-dependent decrease in the DNA signal by UV exposure was also shown.

### 3.3. Long Term Storage and Stability of the Hoechst Reagent for Future Planetary Applications

**Figure 7:**
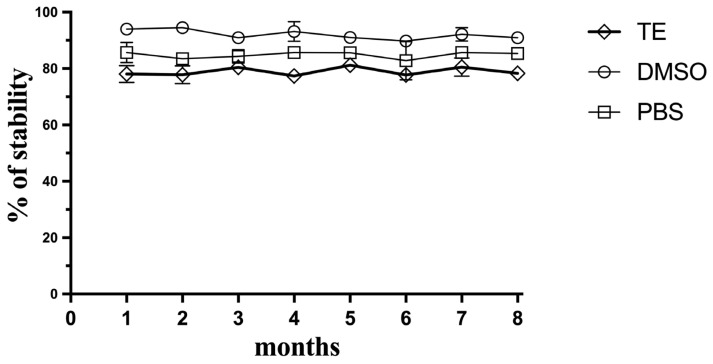
Stability of the Hoechst reagent over storage time. Hoechst was stored as aliquots of the Hoechst reagent frozen in TE buffer, frozen DMSO, or PBS. Data represent average ± SD (N = 3).

Since this method meets the criteria for a potential approach as a planetary exploration kit-based approach, the stability of the Hoechst fluorescent dye during storage was explored over 8 months at −20 °C. The Hoechst dye was mixed with TE buffer, DMSO, or PBS separately, and was then defrosted periodically and compared for its sensitivity with the fresh Hoechst reagent (Figure 7). The dye proved to be stable at −20 °C, especially in DMSO, and retained its ability to detect and quantify DNA in soil simulants for the period of 8 months, which could indicate that this reagent could be prepared in a kit-based approach and sent in a future mission to assay DNA-extracted samples in situ without compromising its sensitivity.

## 4. Discussion

As the essential ingredient for life to form and reproduce, nucleic acids (DNA and RNA) were always considered as potential target molecules in the search for the origins of life. In this context, it is worth noting a 2019 NASA-funded study that synthesized a DNA analogue (hachimoji DNA), incorporating an expanded genetic alphabet that included eight nucleotides instead of the standard four (adenine, cytosine, guanine, and thymine) [25]. This synthetic DNA demonstrated the same core functionalities as its natural counterpart, such as information storage, transmission, and evolutionary adaptability. While this research expands our understanding of what types of molecules could potentially store genetic information in extra-terrestrial life, it also underscores the importance of developing sensitive and versatile detection methods. These methods must be capable of identifying not just canonical DNA, but also its plausible synthetic and extra-terrestrial variants, which our study aims to contribute to.

The detection of DNA on Mars remains an elusive yet tantalizing goal in the search for extraterrestrial life. While numerous missions to Mars have been undertaken over the past several decades, it is important to note that these missions have not specifically aimed to detect DNA on the Martian surface. As a result, the absence of DNA evidence should be interpreted within this context. The primary focus of most Martian missions has been to assess the planet’s geological and atmospheric conditions, with some missions also designed to identify signs of microbial life through other bio-signatures. Our study aims to fill this gap by providing a method that could potentially be adapted for future missions specifically designed to search for DNA or DNA-like molecules. The traditional method for the quantification of DNA relies on UV spectrometry, which can directly detect DNA from samples. It is based on the absorption of nucleotides at 260 nm and can quantify the concentration of DNA, and even for free nucleotides [26]. However, this approach is limited by its range of sensitivity and needs a high concentration of DNA in samples [27]. Compared with UV spectrometry, fluorescent dyes are more sensitive and accurate. Recent research indicated a novel technology called catalysed hairpin assembly (CHA), which can amplify fluorescent signals based on two partially complementary hairpins [28]. For example, thiazole orange (TO) is a cyanine dye and exhibits strong fluorescence when combined with double-stranded DNA. In Zou’s research, TO was embedded in two molecule beacons (MBs) to detect H5N1 DNA with the presence of molybdenum disulfide (MoS_2_) which can lower the background signal due to its high fluorescence quenching ability. This assay can be applied in clinical settings for virus DNA detection with the advantage of low-cost and low-background signals. However, this assay requires the preparation of MoS_2_ nanosheets using the chemical exfoliation method [29] and a reaction is needed to incubate fluorescence measurements at 37 °C for one hour. Another technique involves the use of nanopore sequencing technology, which allows for the real-time sequencing of DNA by passing single-stranded DNA molecules through a nanopore and measuring the changes in an electrical current as each nucleotide passes through the pore [30]. This technology does not require DNA amplification or labeling, which can simplify sample preparation and reduce the risk of sample loss or damage during transportation [31]. Additionally, the small size and light weight of the sequencing instrument make it possible to perform DNA sequencing in remote locations, allowing for the detection of DNA directly on the Martian surface without the need for the sample to return to Earth [32]. However, nanopore sequencing is subject to a noteworthy constraint in that it necessitates high-quality samples containing intact DNA molecules, which may be arduous to obtain from Martian specimens exposed to severe environmental factors such as radiation and oxidation [33].

In this study, we developed a fast and robust method to detect and quantify DNA based on complexes with fluorescent dyes. DNA was spiked onto Martian soil simulants. Subsequently, it was extracted using PBS and then quantified with three different fluorescent dyes: Hoechst, Novel Juice, and PicoGreen. Compared to our assay, all three fluorescent dyes were ready to measure fluorescence fast and provided much quicker alternatives, while there was no requirement to prepare additional chemicals to reduce the background signal. To further evaluate the practicality of our assay, we also investigated the stability of the Hoechst fluorescent dye over an extended period of storage. To this end, the dye was stored at −20 °C for 8 months, and its fluorescence was measured at different time points. Our results suggested that the Hoechst dye retained its fluorescence signal over an 8-month storage period at −20 °C, providing preliminary evidence of its suitability for longer-term storage. This is an important consideration for space exploration missions, where storage conditions and long-duration missions are critical factors. However, it is worth noting that these conditions do not fully mimic those encountered during space missions, where exposure to radiation and potentially longer storage periods may affect dye stability. Future work could aim to evaluate the stability of fluorescent dyes under conditions by more closely approximating those of space missions, including exposure to space-relevant radiation levels. Conversely, the MoS2-based TO assay demonstrated high sensitivity and specificity for H5N1 DNA, but requires some sort of adaptation of molecular beacons to detect different types of DNA. This characteristic makes such methods less flexible when dealing with an undefined spectrum of DNA types, which is a significant consideration for planetary missions where the DNA sequences may be diverse and unfamiliar. In contrast, the assay developed in this study is capable of detecting a broad range of DNA types with high sensitivity. Additionally, electrophoresis was employed to provide visual confirmation of DNA presence.

The surface of Mars is an extreme environment subjected to sub-zero temperatures, harsh oxidizing conditions, strong UV radiation, and a thin carbon-rich atmosphere (95% CO2 occupied), resulting in a limited abundance of life [34]. Nevertheless, sub-surfaces or recently exposed areas offer refuge with reduced radiation exposure, and the possibility of nucleic acids existing in intact forms on the planet has been a topic of interest in recent years. In this study, the impact of UV radiation on DNA stability was evaluated as an initial exploratory effort. The experiment involved 60 min of exposure, which demonstrated the possible detection or persistence of DNA under UV radiation. While these conditions do not completely simulate the Martian environment, and as the implication of a 60-min UV exposure is a foundational step, they serve as an informative first step for assessing DNA degradation under harsh conditions. We recognize the necessity of extended and varied environmental exposure simulations to draw comprehensive conclusions about the potential preservation and detectability of DNA on Mars. The detection of DNA in Martian subsurface areas, where it might be shielded from intense surface radiation or in regions recently exposed due to meteorite impacts or erosion, could provide invaluable insights. These areas could potentially harbour biosignatures, protected from the intense UV radiation and oxidative conditions prevalent on the Martian surface. During Mars’ Noachian age, conditions were ostensibly more conducive to the existence and preservation of life. Therefore, there is a possibility of unearthing preserved biosignatures from this epoch in protected or recently exposed regions [35]. This result is in agreement with the findings of Hansen et al. [36], which showed that cells of cyanobacteria and Bacillus strains can only survive for 3 h maximum under the exposure to the UV radiation. However, by analysing different simulated Martian minerals under the exposure of UV radiation, different minerals imposed different degradation levels of DNA, especially on nucleotides [37]. For instance, labradorite and natrolite can promote the degrading process of nucleotides, whereas apatite, lizardite, and antigorite did not have a degrading effect on nucleotides [37]. Taking the aforementioned into account, these findings could contribute towards the selection of a landing site on future Mars rovers in the search of potential life samples. While our methodology offers a promising approach to DNA detection under simulated Martian conditions, one significant limitation is that we only considered UV radiation in our soil simulants. Mars’s unique soil chemistry also includes oxidizing substances like perchlorates, hydrogen peroxide, and other potentially reactive compounds [38]. These substances could not only further destabilize any DNA present but may also interfere with the fluorescent dyes used in our detection methods. Understanding the full impact of these oxidizing substances on both DNA and fluorescent dyes would require additional testing and, possibly, modifications to the detection methods. These complexities should be considered in future research to make this method more applicable to real-world astrobiology missions.

While our method provides a potentially more sensitive and versatile approach for DNA detection, it is crucial to note the possible issue of Earth-based contamination [39]. In an astrobiology context, especially in missions targeting the search for extra-terrestrial DNA or DNA-like molecules on planets like Mars, minimizing the risk of Earth-based contamination is a fundamental concern. Contamination from Earth could pose a serious challenge to the ‘classical laboratory techniques’ employed in these missions, potentially skewing the results and interpretations [40]. Therefore, while our study paves the way for more sensitive DNA detection methods, the issue of contamination needs to be addressed meticulously in future missions. This could include sterile procedures for sample collection, specialized containment units for sample storage, and rigorous protocols for distinguishing between Earth-based and potentially Martian genetic material.

In conclusion, the search for DNA on Mars is a challenging but exciting endeavor that has the potential to provide invaluable insights into the possibility of extraterrestrial life. While the detection of intact DNA in Martian samples remains a significant challenge, ongoing research and advancements in technology hold promise for future missions. The development of novel detection methods, such as fluorescent dyes and nanopore sequencing, are paving the way for more sensitive and robust approaches to help detect and quantify DNA in complex samples. With advancements in miniaturized spectrophotometers and fluorescence detectors, it is feasible to integrate such a system into a rover’s suite of analytical tools. The operations can be automated, reducing the need for human intervention and allowing for continuous data collection and transmission. As we continue to push the boundaries of our understanding of the universe, the search for DNA on Mars will undoubtedly remain at the forefront of astrobiological research and exploration. The adaptability of our DNA detection protocol for Martian rover integration holds potential for future Mars exploration missions. While challenges exist, they are surmountable with current technological advancements. The incorporation of such a method could be pivotal in our search for signs of life on the Red Planet.

## Data Availability

Data from gel analyses were provided following the submisison. Any other data will be available on request.
